# Exploiting phage-antibiotic synergies to disrupt *Pseudomonas aeruginosa* PAO1 biofilms in the context of orthopedic infections

**DOI:** 10.1128/spectrum.03219-23

**Published:** 2023-12-12

**Authors:** Steven De Soir, Hortence Parée, Nur Hidayatul Nazirah Kamarudin, Jeroen Wagemans, Rob Lavigne, Annabel Braem, Maya Merabishvili, Daniel De Vos, Jean-Paul Pirnay, Françoise Van Bambeke

**Affiliations:** 1 Pharmacologie cellulaire et moléculaire, Louvain Drug Research Institute, Université catholique de Louvain, Brussels, Belgium; 2 Laboratory for Molecular and Cellular Technology (LabMCT), Queen Astrid Military Hospital, Neder-over-Heembeek, Belgium; 3 Department of Materials Engineering, Biomaterials and Tissue Engineering Research Group, KU Leuven, Leuven, Belgium; 4 Department of Chemical and Process Engineering, Faculty of Engineering and Built Environment, Universiti Kebangsaan Malaysia, Bangi, Selangor, Malaysia; 5 Laboratory of Gene Technology, KU Leuven, Leuven, Belgium; Innovations Therapeutiques et Resistances, Toulouse, France

**Keywords:** bacteriophage therapy, antibiotics, *Pseudomonas aeruginosa*, biofilms

## Abstract

In recent years, bacteriophages (or phages) have reentered the spotlight as alternative or adjuvant therapeutic agents to antibiotics. Their efficacy in more recalcitrant forms of infections like biofilms, frequently encountered in the orthopedic setting, remains less characterized. The present study aimed at evaluating the activity of phage-antibiotic combinations against *Pseudomonas aeruginosa* biofilms. A large collection of phages was *de novo* isolated from a wide variety of environmental sources. Three phages with a large host range against a.o. clinical orthopedic isolates, PSP2 (*Yuavirus*), PSP3 (*Pbunavirus*), and PSP30 (*Bruynoghevirus*), were selected for phage-antibiotic synergy assays on mature *P. aeruginosa* PAO1 biofilms. Phages were combined with ciprofloxacin, meropenem, and ceftazidime, all used as standard of care in bone-related infections. Significant reductions in both cell counts and biomass were observed for all phage-antibiotic combinations. The highest reduction in viable cell counts was observed for PSP3 and PSP2 in combination with ceftazidime [4.58 and 4.30 log_10_ Colony Forming Units (CFU)/well], followed by all phage combinations with ciprofloxacin (up to 3.56 log_10_ CFU/well). The highest reduction in biomass was obtained by combining PSP3 with ciprofloxacin (29.8%). Metabolic assays confirmed these findings with reductions in biofilm respiratory rate of up to 65%. Scanning electron microscopy imaging of PAO1 biofilms grown on titanium coupons and treated by ciprofloxacin and PSP30 confirmed the efficacy of the combined treatment with PSP30 and ciprofloxacin. This study highlights the synergetic effects of phage-antibiotic combinations on *P. aeruginosa* biofilms, thereby offering a promising approach to combat biofilm-associated infections.

**IMPORTANCE:**

Biofilm-related infections are among the most difficult-to-treat infections in all fields of medicine due to their antibiotic tolerance and persistent character. In the field of orthopedics, these biofilms often lead to therapeutic failure of medical implantable devices and urgently need novel treatment strategies. This forthcoming article aims to explore the dynamic interplay between newly isolated bacteriophages and routinely used antibiotics and clearly indicates synergetic patterns when used as a dual treatment modality. Biofilms were drastically more reduced when both active agents were combined, thereby providing additional evidence that phage-antibiotic combinations lead to synergism and could potentially improve clinical outcome for affected patients.

## INTRODUCTION

Bacteriophages are ubiquitous viruses that target, infect, and potentially kill only bacterial cells with very high species specificity, thereby leaving off-target bacteria unharmed, unlike most other antimicrobial substances (1). Their discovery already dates back to the early 20th century, but phage research and phage therapy lost their attraction due to the discovery of penicillin and the subsequent construction of an entire pharma industry producing static chemical compounds (2). Although Fleming already warned the scientific world in his Nobel Prize winning lecture that he himself observed bacterial cultures quickly becoming resistant to this newly found antibiotic, Western Society neglected his concerns while continuously misusing antibiotics, thereby leading toward the antibiotic crisis experienced today (3). New pan-drug-resistant bacterial strains are emerging on a daily basis, becoming a huge threat to human life with estimates of over 10 million deaths annually by the year 2050 and an economic burden estimated to surpass 100 trillion USD, if we do not start to act and elicit a paradigm shift immediately (4).

On top of this alarming acquisition of resistance, bacteria can also adopt specific lifestyles, making them less responsive toward antibiotic treatment. Among them, biofilms are the most prevalent mode of life for bacterial populations and are defined as a community of micro-organisms embedded in, and protected by, a self-produced extracellular matrix composed of lipopolysaccharides, cellular debris, extracellular DNA (eDNA), proteins, and teichoic acid (5, 6). Most often, these biofilms adhere on biotic or abiotic surfaces. During biofilm formation, bacterial cells undergo a metabolic shift, coordinated by quorum sensing and due, in part, to the limited availability of nutrients or oxygen, resulting in “dormant” or metabolically inactive cells. Most of the currently used antibiotics target active, metabolic pathways, as is the case for beta-lactams or fluoroquinolones (inhibiting cell wall synthesis and DNA replication, respectively) (7). These compounds will thus lose their target and efficacy, making bacteria embedded in biofilms 500–5,000 times more tolerant toward these antibiotics (8). A staggering 80% of all clinical bacterial infections are complicated due to a biofilm component, of which at least 20% are polymicrobial (a widely underestimated number) (9).

For orthopedic infections and peri-prosthetic joint infections (PJIs) in particular, the prevalence of biofilm-related infections is relatively low. However, bacteria can adhere up to 10,000 times better to the implant material than the native tissue surrounding this implant (8). Once bacteria have colonized the implant material, failure rates of current antibiotic therapies are extremely high (up to 90%) (10). Depending on the source, 1%–5% of all arthroplasties lead to an infected implant with re-occurrence of the infection in almost 40% of affected patients (11 – 14). For almost 70% of these patients, members of the *Staphylococcus* genus are the leading causative agent (predominantly coagulase negative staphylococci such as *Staphylococcus epidermidis* account for more than 30%, while coagulase positive *Staphylococcus aureus* is present in over 20%–25% of the cases) (7). Notwithstanding the relatively low occurrence of *Pseudomonas aeruginosa* when compared to the previously named Gram-positive bacteria, it is the causative pathogen in about 20%–30% of PJIs caused by Gram-negative bacteria (15). In view of its frequent resistance to multiple classes of antibiotics, these infections are extremely difficult to treat and markedly affect the quality of life of the affected patient (15, 16). New treatment options and novel strategies are therefore urgently needed.

Current treatment strategies consist of debridement followed by aggressive and prolonged antibiotic treatment, i.e., salvage debridement, antibiotics, and implant retention. However, as mentioned before, failures are extremely frequent, and replacing the implant with a novel one is a delicate procedure, especially when bacterial cells remain present in the peri-prosthetic area, even after this excessive antibiotic treatment. When opening the wound and replacing the implant, a new window for surgical site infections is created, thus initiating a cycle of infection once again. A promising alternative could be the combined use of phages and antibiotics. Phages have long been shown to possess depolymerase activity on biofilm matrix components, thereby disrupting biofilm structure while prompting these biofilm bacteria to shift their metabolic state from a dormant one to an active planktonic one, making the metabolic target available for antibiotics (17). The present study aims to investigate the synergy between routinely used antibiotics and *de novo* isolated phages for the disruption of mature biofilms formed by *P. aeruginosa* strain PAO1, including on titanium coupons mimicking the material used in orthopedic implants.

## RESULTS

### *De novo* isolation and characterization of phages from a wide variety of sampling sources

The isolation and purification process resulted in the collection of 67 *P*. *aeruginosa* phage clones isolated from a wide variety of environmental samples (Table S1). This phage collection was screened against 32 *P*. *aeruginosa* strains [including 11 clinical orthopedic strains, 2 phage production strains (PAO1 and CN573), and 19 strains from the LabMCT collection that represent more than 10 genetically different strains as determined by AFLP typing (18)] to define host range of each individual phage (Table S2 and S3). There was not one bacterial strain for which no lytic phage activity was observed (Table S3). From this collection, 20 phages with lytic activity against *P. aeruginosa* strain PAO1 in solution were selected and first pre-screened on PAO1 biofilms. Three phages, PSP2, PSP3, and PSP30, with broad host range and showing highest anti-biofilm activity as determined by biomass and Colony Forming Units (CFU) evaluations in preliminary work, were selected for phage-antibiotic synergy (PAS) assays.

These three phages were further characterized. Based on their genome sequence, they were identified as highly similar to members of the genus *Yuavirus* (family Mesyanzhinovviridae) for phage PSP2, genus *Pbunavirus* (family Myoviridae) for phage PSP3, and belonging to genus *Bruynoghevirus* (family Podoviridae) for phage PSP30.

The most important genomic characteristics of all three phages are summarized in Table 1. Multiple tools were used and could not confirm the presence of depolymerase genes as no pectate lyase domains were identified in the positive hits generated by PhageDPO or DePP in the genomes of these three phages (nor were additional hydrolases or lyase domains identified which are often found in phage-encoded depolymerases active on *P. aeruginosa* (19). Sequence alignments of presented phages can be found in Fig. S1.

**TABLE 1:** Summary of genomic characteristics of phages PSP2, PSP3, and PSP30

	PSP2	PSP3	PSP30
Genome size (bp)	61,810	66,308	45,373
Identification (% identity ***)***	Yuavirus, Siphoviridae (96.06%)	Pbunavirus, Myoviridae (96.01%)	Bruynoghevirus, Podoviridae (96.03%)
Coding sequences	89	94	69
Protein-encoding genes with functional assignment	51	45	67
Protein-encoding genes without functional assignment	38	49	2
% Protein-encoding feature coverage	143.99	141.76	152.07
% Features that are hypothetical	42.7	52.13	2.9

### PAS assays

#### Biomass and viable cell counts

**Fig 1:**
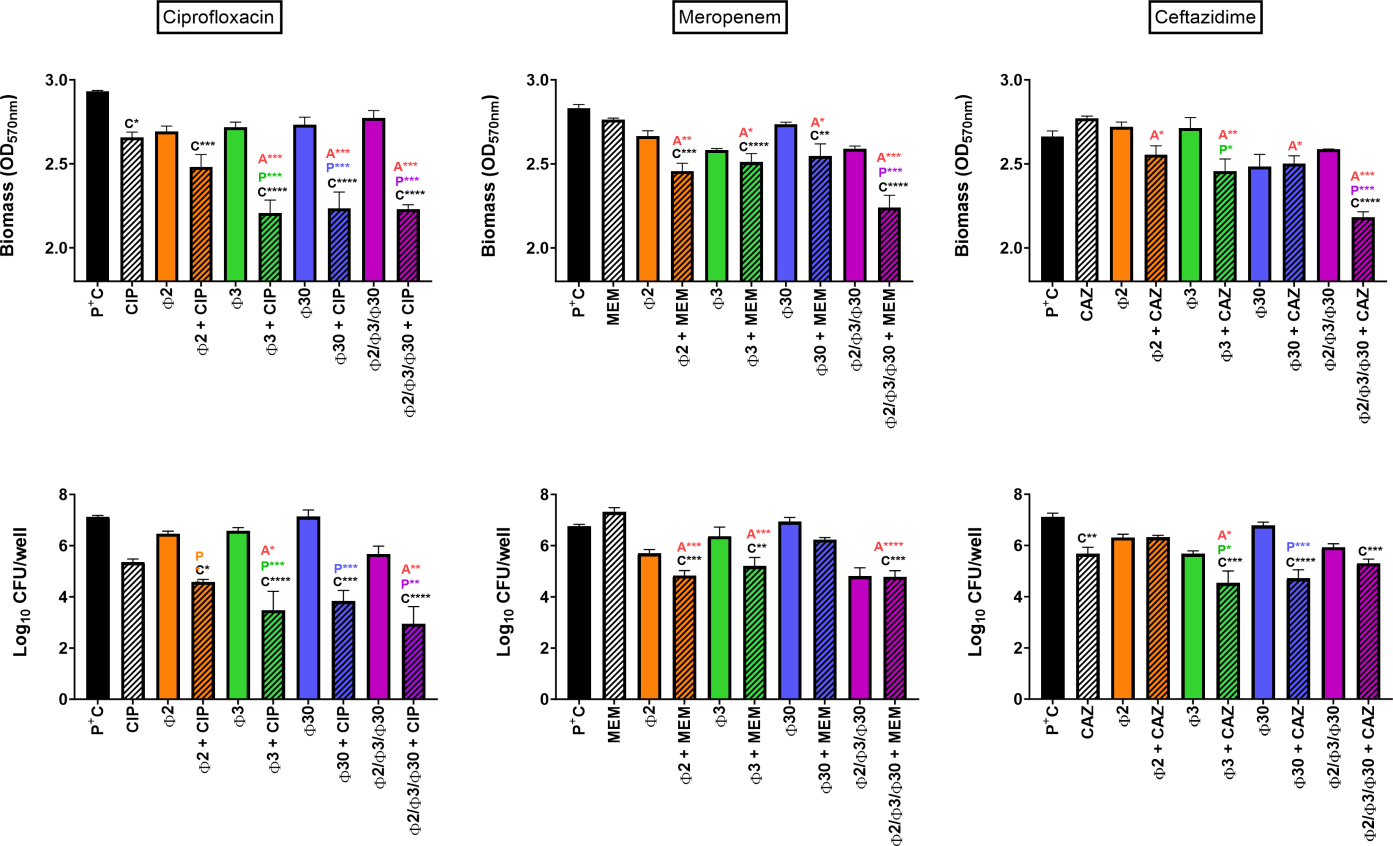
Activity of antibiotics and phages against mature 24 h-biofilms formed by *P. aeruginosa* PAO1. Biofilms were incubated during 24 h with antibiotics at 1× MIC [ciprofloxacin (CIP; left), meropenem (MEM; middle), and ceftazidime (CAZ; right)], phages at 10^9^ PFU/mL (Φ2 = Phage PSP2; Φ3 = PSP3; Φ30 = PSP30) or their combinations. Top: residual biomass, evaluated by OD_570nm_ of crystal violet; bottom: viable cell counts expressed in Log_10_ CFU/well. Statistics: One-way ANOVA analysis with Tukey post-test (statistically different from positive control = C, from phage alone = *P*, and from antibiotic alone = A with *P* < 0.05 = *, *P* < 0.01 = **, *P* < 0.001 = ***, and *P* < 0.0001 = ****). Data are mean ± SEM (*N* = 3; *n* > 6).

In the first set of experiments, mature 24 h-biofilms grown in 96-well plates were incubated for 24 h with antibiotics at a concentration of 1× MIC, phages PSP2, PSP3, and PSP30 individually or in a cocktail [each at 10^9^ Plaque Forming Units (PFU)/mL] or a combination of each antibiotic with phages, after which residual biomass and CFU were measured (Fig. 1).

Considering residual biomass first, ciprofloxacin significantly reduced it by 9.3%, whereas meropenem and ceftazidime proved ineffective with a reduction of 2.4% and even a 4% increase in biomass, respectively. Individual phages or the phage cocktail did not cause any significant reduction in biomass. However, when phage-antibiotic combinations were applied to the biofilms, there was a significant reduction of biomass. Combinations of ciprofloxacin with individual phages and the phage cocktail showed higher biomass reduction compared to ciprofloxacin alone (up to 15.3%, 24.7%; 23.7%, and 23.9%, for PSP2, PSP3, PSP30, and the cocktail, respectively). Similarly, the combinations of all three phages or the cocktail with meropenem or ceftazidime resulted in a higher reduction in biomass compared to the antibiotics alone. The maximal biomass reduction achieved with these combinations was ranging from 18% with ceftazidime, 20.9% with meropenem, and 24.7% with ciprofloxacin.

Considering the residual CFU counts, ciprofloxacin was the most active antibiotic, causing an average reduction of 1.71 log_10_ CFU/well, followed by ceftazidime, which decreased CFU by 1.36 log_10_/well. Meropenem slightly increased bacterial counts (on average 0.61 log_10_ CFU/well), but this effect was not statistically significant. Individual phages did not cause significant reductions in CFU, reaching from 0.87 log_10_ CFU/well for phage PSP2 (the most active phage), while the phage cocktail caused a marginal (1.41 log_10_ CFU/well), but still not significant effect. In contrast, when phages were combined with antibiotics, reductions in cultivable cells were significantly larger than seen for phages or antibiotics alone. Specifically, the combination with ciprofloxacin proved to be very efficient with reductions ranging from 2.48 log_10_, 2.69 log_10_, and 2.97 log_10_ CFU/well with individual phages PSP2, PSP3, and PSP30 and this fluoroquinolone. With meropenem, phage PSP2 was shown to be the most effective (reduction of 1.85 log_10_ CFU/well), while phages PSP3 and PSP30 showed the highest synergetic interaction for the combination with ceftazidime (reduction of 2.32 log_10_ CFU/well), resulting in significantly higher activity than either of these antibiotics alone. The phage cocktail was generally not more effective than the most active individual phage for a given phage-antibiotic combination. Overall, in combination with the phage cocktail, ciprofloxacin was the most effective antibiotic with a reduction of 3.64 log_10_ CFU/well, vs 1.88 log_10_ CFU/well for meropenem, and 1.79 log_10_ CFU/well for ceftazidime.

Globally, when defining synergy as a greater effect for the combined treatment than the sum of the effects of its individual components (20), we observed synergy between the antibiotics and all phages, except for PSP2 with ciprofloxacin to reduce biomass, and PSP2 and PSP3 with ceftazidime to reduce viability.

**Fig 2:**
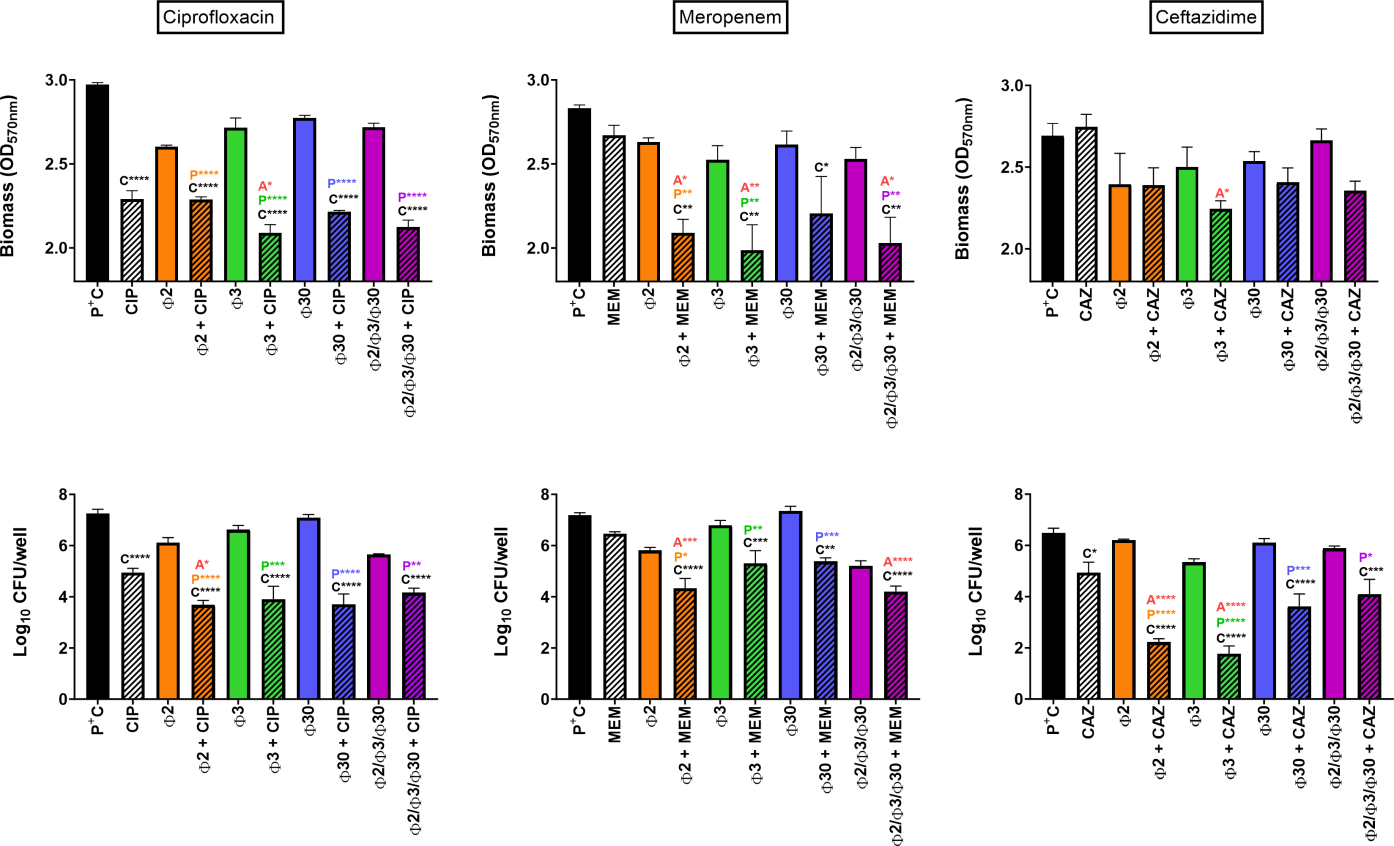
Activity of antibiotics and phages against mature 24-h biofilms. Biofilms were incubated during 24 h with antibiotics at 10× MIC [ciprofloxacin (CIP; left), meropenem (MEM; middle) and ceftazidime (CAZ; right)], phages at 10^9^ PFU/mL (Φ2 = Phage PSP2; Φ3 = PSP3; Φ30 = PSP30) or their combinations. Top: residual biomass, evaluated by OD_570nm_ of crystal violet; bottom: viable cell counts expressed in Log_10_ CFU/well. Statistics: One-way ANOVA analysis with Tukey post-test (statistically different from positive control = C, from phage alone = *P*, and from antibiotic alone = A with *P* < 0.05 = *, *P* < 0.01 = **, *P* < 0.001 = ***, and *P* < 0.0001 = ****). Data are mean ± SEM (*N* = 3; *n* > 6).

These experiments were repeated by retaining a fixed concentration of phages at 10^9^ PFU/mL, while increasing antibiotic concentrations to 10× MIC (Fig. 2). This resulted in an improved biofilm eradication for both biomass and cultivable bacterial cells. Considering the biomass, ciprofloxacin remained the only antibiotic capable of significantly reducing it when used alone (up to 22.9% decrease). All combinations with phages except for PSP2 proved slightly more active, resulting in a biomass decrease of 25%, 28.5%, and 29.7% for PSP30, the phage cocktail, and PSP3, respectively. The improvement was significant only when ciprofloxacin was combined with PSP3. PSP3 showed the highest interaction with antibiotics at these concentrations and significantly enhanced the effect of meropenem and ceftazidime, leading to biomass reductions of 29.8% and 16.6%, respectively. PSP2 improved the effect of meropenem only (26.2% decrease). Regarding viable counts, combined treatments were more active than antibiotics or phages alone. With ciprofloxacin, the maximal reductions in CFU were similar to those observed for combinations of phages with the antibiotic at 1× MIC, ranging from 2.91 to 3.55 log_10_ CFU/well reductions with individual phages and log_10_ 3.08 CFU/well reductions using the phage cocktail as an adjuvant. A gain in activity was also observed when phages were combined with beta-lactams at 10× the MIC. PSP2 was seen to be the most active phage with meropenem (2.63 log_10_ CFU/well reduction), and PSP3 showed the highest interaction with ceftazidime (4.58 log_10_ CFU/well). When combined with the phage cocktail, meropenem and ceftazidime achieved reductions of 2.89 and 1.92 log_10_ CFU/well, respectively. The phage cocktail was thus not more active than the most active individual phage (and was even less active when combined with ceftazidime).

Globally, to reduce biomass, all phage-antibiotic combinations with meropenem yielded synergetic results as well as the combination of PSP3 with ceftazidime, while for CFU counts, all combinations with meropenem and ceftazidime (except for the phage cocktail) indicated synergetic patterns. Although synergism couldn’t always be attained with ciprofloxacin, the combined treatment always showed higher reductions for both biomass and CFU when compared to any type of agent alone, except for the combination with PSP2. A full list of all additive and synergetic profiles is provided in supplementary materials (Tables S4 through S6).

#### Omnilog assays

**Fig 3:**
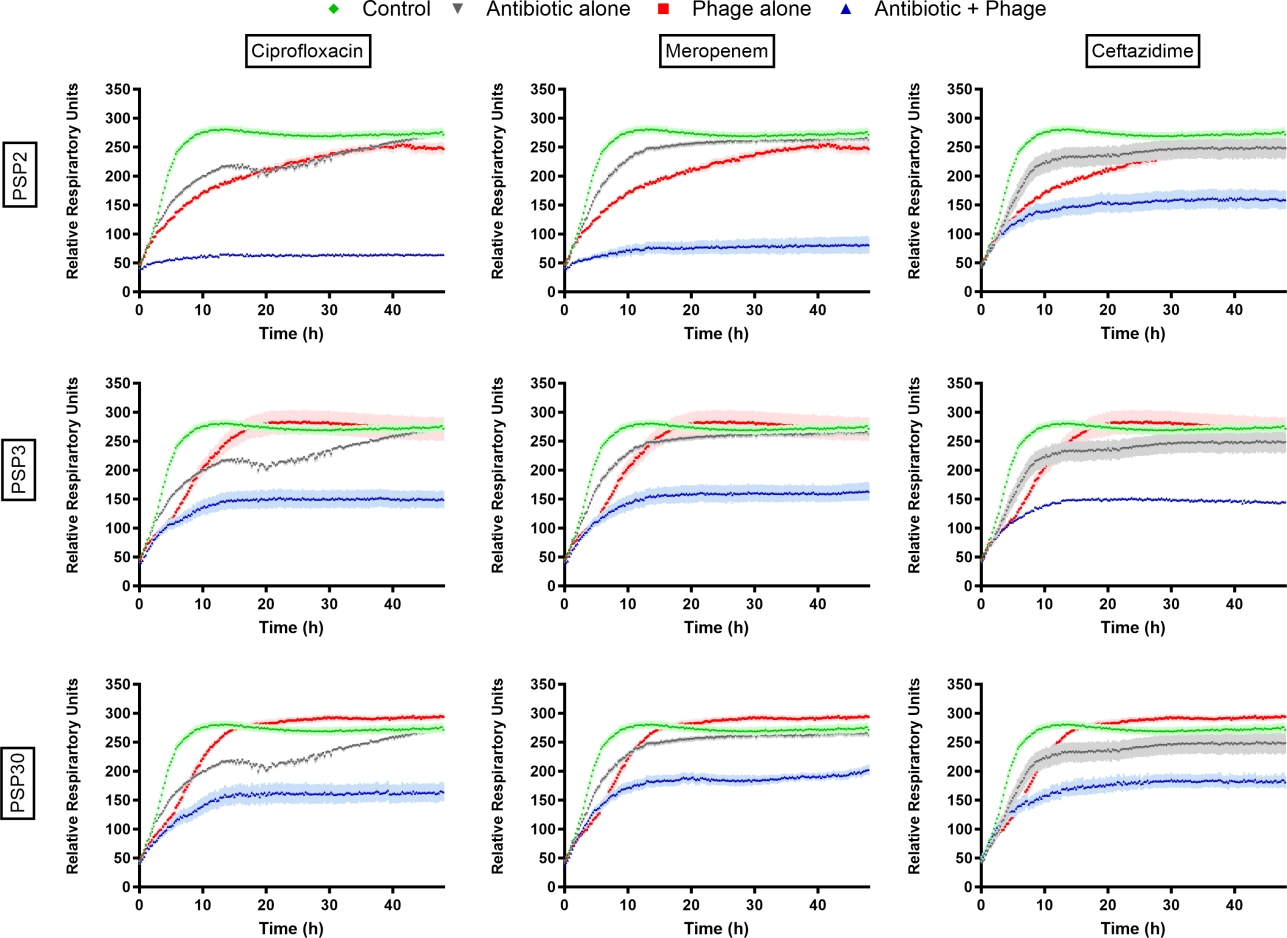
Respiratory activity in 24 h biofilms, assessed by the metabolization of a tetrazolium dye in the OmniLog over 48 h of culture in control conditions or in the presence of 10^9^ PFU/mL of phages PSP2 (Φ2, top), PSP3 (Φ3, middle), and PSP30 (Φ30, bottom), antibiotics at 10× MIC [ciprofloxacin (left), meropenem (middle), and ceftazidime (right)] or their combinations. Data are means ± SEM (*N* = 3; *n* = 6).

In parallel to previous experiments, metabolic activity in biofilms was tracked over time by measuring the respiration rate of the bacterial cells present in the biofilm, as determined by the degradation of a tetrazolium dye using the OmniLog system (Fig. 3). These measurements confirmed previous findings showing that in general, the combination of phages and antibiotics was more efficient than individual treatments. When phages (10^9^ PFU/mL) or antibiotics (10× MIC) were used individually, a delay in the metabolization of the dye was observed. However, after 48 h of exposure, the same plateau levels were achieved as for the untreated biofilms. When phages were combined with antibiotics, drastic reductions in the biofilm respiratory rate were seen immediately after their addition and were maintained throughout the 48 h of exposure. As a result, the plateau levels for the combined treatments were more than 65% lower when compared to monotherapies or untreated biofilms. The highest reductions were observed for the combination of PSP2 with ciprofloxacin and meropenem, while PSP30 was the least synergetic.

#### Scanning electron microscopy

**Fig 4:**
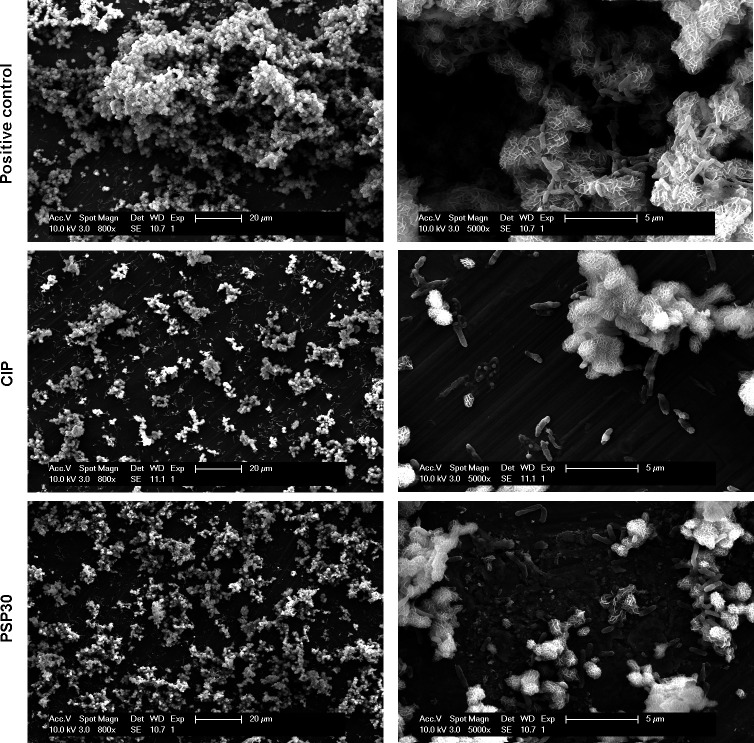
Images in SEM of 48 h biofilms grown on titanium coupons, in control conditions (top) or exposed to ciprofloxacin (CIP) at 100× MIC (middle) or 10^7^ PFU/mL of phage PSP30 (bottom). Bars: 20 µm (left) and 5 µm (right).

**Fig 5:**
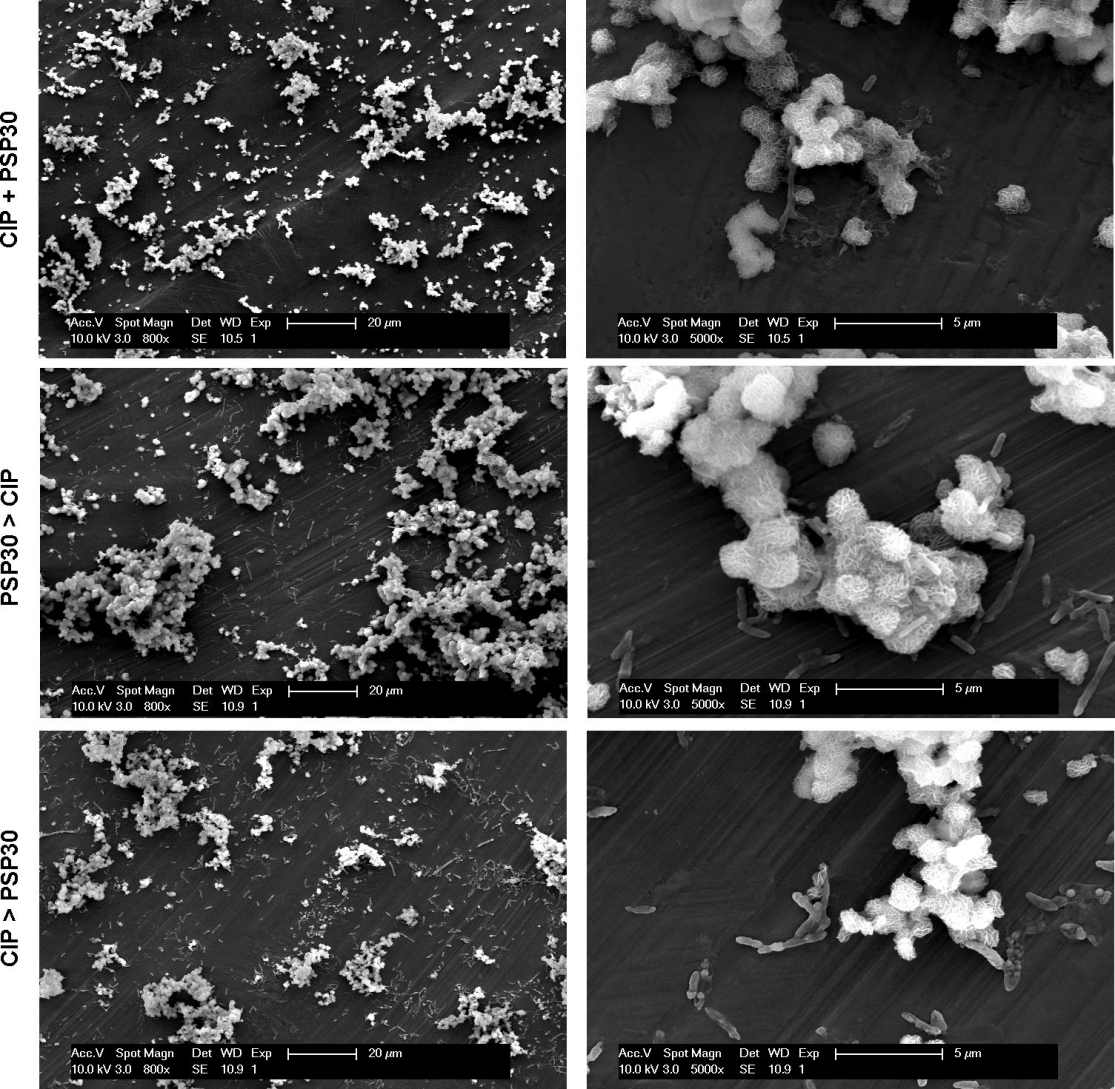
Images in SEM of 24 h biofilms grown on titanium coupons and exposed to ciprofloxacin (CIP) at 100× MIC and 10^7^ PFU/mL of phage PSP30 concomitantly during 48 h (top) or sequentially (24 h each), starting by the phage (middle) or by the antibiotic (bottom). Bars: 20 µm (left) and 5 µm (right).

To visualize the effects of phage-antibiotic combinations on biofilms, control and treated biofilms were examined using scanning electron microscopy (SEM). In a translational perspective, biofilms were grown on titanium coupons (material used in orthopedic implants) and exposed to ciprofloxacin (most active drug in this study) at a concentration of 100× MIC close to the concentration reached in the bones of treated patients [14.6 mg/L (21)]. Phage PSP30 was selected based on a previous study (22), suggesting that *Bruynoghevirus* could be appropriate for therapy, due to their strictly lytic cycle and the absence of virulence factors or antibiotic resistance determinants and allergens. It was added at 10^7^ PFU/mL [inoculum generally used in the clinics (23)]. The combined treatment with the antibiotic and the phage was also compared to sequential treatment, in which the phage was applied for 24 h, followed by the antibiotic for another 24 h, or vice versa. Incubation time was extended to 48 h for all other conditions. Fig. 4 shows typical images of control biofilms and biofilms exposed to ciprofloxacin or to PSP30 individually. In control biofilms, voluminous aggregates covering most of the surface of the coupon were observed, with a few isolated bacteria adhering to the coupon surface. At higher magnification, these aggregates appeared to be resembling the crystal structure of sand desert roses, with a few bacteria also adhering to it. In biofilms exposed to ciprofloxacin, aggregates appear much smaller and bacteria remained visible on the coupon surface. At higher magnification, many isolated bacteria were distorted and harbored large vesicles on their surface. In biofilms exposed to PSP30, the image at higher magnification revealed the presence of undefined material deposited at the surface of the coupons and of bacteria emerging from the aggregates. Fig. 5 shows typical images of biofilms exposed to ciprofloxacin and PSP30 concomitantly or sequentially. When the combined treatment was applied, the coupon’s surface was almost entirely clean, without any residing bacteria, but with the sequential treatments, small aggregates remained present in all cases. Distorted, vesiculated bacteria were visible for all conditions including ciprofloxacin. Additional images can be found in Fig. S2 and S3**.**

## DISCUSSION

This work shows that the combined use of phages with ciprofloxacin, meropenem, or ceftazidime is in general more effective in eradicating *P. aeruginosa* PAO1 biofilms *in vitro* than any of these agents individually.

The degree of synergy is variable among the antibiotics, depending on their own activity: with ciprofloxacin, the most active antibiotic when tested alone, synergy is best seen at the lowest antibiotic concentration (1× MIC), while an additive effect is rather obtained at higher concentration (10× MIC). Conversely, with ceftazidime, which is less active in monotherapy, synergistic reduction of bacterial counts is essentially observed at 10× MIC. For meropenem, which shows an intermediate activity, the benefit of phage addition is clearly seen at both concentrations. This is in line with previous studies, showing that the benefit of phages is best observed in conditions where the activity of antibiotics is suboptimal (24, 25). This may have important implications *in vivo*, where antibiotic concentrations fluctuate over time.

As biofilms are the prevailing mode of life for bacterial populations, phages have evolved mechanisms to co-exist in this micro-environment (17). A lot of advances have been made in recent years in deciphering and understanding the effects of phage-produced enzymes, such as endolysins (26) and depolymerases (27, 28) on biofilm matrix components, in addition to the effects of whole phage therapy on biofilms. Genes encoding depolymerases were not identified in the phages used here, but still, synergistic interactions were observed, as was also the case in the work of Akturk et al*.* (24). The mechanism of this synergy therefore needs to be further explored. Several possible mechanisms of antibiotic-phage synergy have been proposed (29) but not specifically studied in biofilms. Akturk et al. propose that phages can get access to the bottom of the biofilm via void spaces. If such is the case, they could potentially re-activate bacterial metabolism by an increased pressure applied on biofilms when multiple active agents are used, thereby increasing possible target sites for antibiotics as well as virulent phage production (30).

A recent study identified a newly isolated representative of genus *Bruynoghevirus*, similar to our phage PSP30, as a promising alternative treatment (31), while others identified *Yuavirus* members, amongst others, as efficient anti-biofilm reducers (31, 32), but their pseudolysogenic character is still a matter of debate (33). However, many of these studies investigate the inhibition of biofilm formation rather than mature biofilm removal.

When looking at combined treatment modalities, the concept of PAS is not new. The first study introducing the term PAS was published in 2007 by Comeau et al*.* (34). They observed that, by combining phages with sublethal concentrations of antibiotics, including cephalosporins and quinolones, they could stimulate the production of virulent particles produced by host strains such as *Escherichia coli*. This effect was seen to be SOS-independent [i.e. independent of an induction of proteins and error-prone factors promoting DNA integrity, improved survival and continuous DNA replication in situations of severe DNA damage (35)], related to cellular filamentation, and shared between different genera of phages. In the following years, similar synergetic effects between phages and different types of antibiotics could be observed for other species, including foodborne pathogens (36), *Burkholderia cenocepacia* (37)*, Klebsiella pneumoniae* (38), *S. aureus* (39 – 47), *Acinetobacter baumannii* (48 – 51), *Enterococcus* spp. (*faecium* and *faecalis*) (52 – 55), and *P. aeruginosa* (24, 25, 56 – 65). However, only a limited number of these studies have been performed on biofilms.

In 2018, Uchiyama et al*.* reported synergetic profiles of five genetically different phages in combination with 25 distinct antibiotics on different *P. aeruginosa* strains including PAO1. The phages evaluated in their work belonged to different genera, but interestingly, the strongest synergetic effects were observed for *Pbunavirus* members (56). Although these authors did not investigate PAS patterns on mature biofilms, they did observe inhibition of *P. aeruginosa* PAO1 biofilm formation by phage-antibiotic combinations (56). More recent studies confirmed the activity of phages from the *Pbunavirus* genus on *P. aeruginosa* biofilms (66, 67). In the context of cystic fibrosis, PAS was also observed for nebulized phage PEV20 and ciprofloxacin, not only on planktonic cultures of clinical *P. aeruginosa* strains but also on the formation and growth of biofilms, with the combined treatment being more efficient than any of these agents alone, in line with our own observations made with ciprofloxacin and phages PSP2, 3, and 30 (25, 58).

While previous studies only evaluated the combined administration of phages and antibiotics, Chaudry et al. were the first to report on the effect of the sequential treatment modalities (65). In their hands, biofilm eradication was higher when phages were administered before antibiotics acting on protein synthesis (aminoglycosides) and to a marginal extent with fluoroquinolones, while simultaneous combination was more effective with a cephalosporin. This is consistent with our observations made for PAO1 biofilms grown on titanium coupons and visualized post-treatment with SEM, which did not evidence major differences between sequential and combined treatment modalities. We only noticed that the least bacterial cells were seen residing on titanium coupons when phage PSP30 was combined for 48 h with ciprofloxacin. Another interesting observation made on SEM images in our work concerns the appearance of vesicular material at the surface of bacteria exposed to ciprofloxacin. These vesicles are resembling the outer membrane vesicles described in *E. coli* and *P. aeruginosa* planktonic cultures exposed to ciprofloxacin. Some studies attribute their formation to the SOS response induced by this drug (68, 69). These vesicles may reflect a delay in cell division and alterations of the envelope integrity, eventually leading to cell death. Others rather suggest they are the hallmark of an explosive cell lysis due to an endolysin-encoding gene located on the genome within a cryptic prophage gene cluster that encodes the R- and F-type pyocin (70). This lysis would liberate membrane vesicles as well as proteins and eDNA serving as public goods in biofilms.

In general, the effect of the cocktail of phages is of the same order of magnitude as the effect observed for the most active phage, i.e., PSP3, in most of the cases. Although higher activity for phage cocktails than individual phages is generally observed (71), antagonism or absence of synergy between phages in a closed, in-vitro system, such as a biofilm, has already been described (72 – 75) and could be due to a competition for the same host, especially if they interfere with different metabolic pathways (76). From an evolutionary perspective, this host competition results in one phage becoming the dominant one in the population, overtaking the less-performing ones, hence the observed effect over time. However, this never led to antagonism in our short-term experiments. This is of importance, as the use of phage cocktails remains of interest *in vivo*. Before tailored phage therapy could be applied, phage cocktails remain the most effective short-time treatment as they increase the host range that can be targeted (77 – 79) and prevent or delay the selection of resistance (72, 80). However, evolutionary trade-offs occurring in bacterial populations after long-term phage therapy are more difficult to predict when applying a wide variety of phages all at once. Recently, Chan et al*.* showed that, by applying personalized phage therapy, they could decrease virulence and effectively re-sensitize *P. aeruginosa* strains against multiple antibiotics (81). By twisting evolution in their favor, they could improve antibiotic treatment and therapeutic outcome further underlining the importance of a phage-antibiotic combination therapy, especially in a patient-tailored approach. For the phages in this work, bacterial receptors were not found to be related to any kind of antibiotic efflux proteins. Literature suggests they could adhere either to the LPS (PSP3 and PSP30) (82), the non-contractile host pili (PSP2) (83), or the type IV pili (PSP30) (82). Resistance toward these phages would therefore not necessarily result in higher susceptibility to antibiotics but rather in decreased bacterial motility and virulence.

Quantification of biofilm biomass is often performed using crystal violet staining, followed by optical density measurements. This technique is rapid but does not discriminate between live and dead cells or extracellular matrix. Counting of colonies gives a more accurate estimation of the viable bacteria that keep their replicative capacities in the biofilm. Yet, this method is laborious and gives only endpoint measurements (84). The assay set up here using the OmniLog system to follow in real time the metabolic activity in the biofilms allows to detect early on synergies between phages and antibiotics, focusing on the treatment effects on viable bacteria. This type of metabolic assay could be used in the future after full validation for the high throughput screening of active phage-antibiotic combinations or of any other type of innovative treatment. A limitation of this technique, however, is that the added dye accumulates and is not re-metabolized, meaning that a decrease in metabolic activity will not be detectable once a plateau is reached. It therefore still needs to be combined with the evaluation of biomass, and CFU residual counts for a global picture of treatment effects on biofilms (85).

Our work presents a series of additional limitations. First, we tested a single reference strain (PAO1), which does not represent the diversity faced in the clinics, including the capacity of individual strains to form biofilms. This is partially mitigated by the fact that we determined a wide host range for the phages selected for our study. The use of a susceptible, standard strain was important as a starting point for such a study in order the set-up the scene before working with clinical isolates. Second, our experiments were limited to *in vitro* models, but this is the first indispensable step to set-up optimal conditions for further preclinical evaluations. Third, phages were not pre-screened genetically for the identification of possible biofilm-degrading enzymes, which could have helped to the selection of biofilm-active phages. Yet, although huge efforts have been made to identify depolymerases, etc. in the genome of phages, annotations, and protein identifications are often limited with a huge part of the “phageome” being classified as hypothetical proteins with its functions still remaining to be elucidated, leaving thus room for general screening, as done here. Fourth, no phage-bacterial evolution patterns were evaluated as treatment protocols only consisted of daily treatments. With orthopedic patients often presenting long-term recurrent infections, the effect of prolonged exposure to phage-antibiotic combinations should be meticulously investigated.

To conclude, the present study highlights the synergetic effects of phage-antibiotic treatment modalities on *P. aeruginosa* PAO1 biofilms, thereby offering a promising approach for combating biofilm-associated infections. Future efforts are warranted to explore the mechanisms underlying these synergetic interactions and evaluate their potential in a clinical setting.

## MATERIALS AND METHODS

### *De novo* isolation and characterization of phages active against *P. aeruginosa*

Eleven *P. aeruginosa* strains were collected from the orthopedic ward at the *cliniques universitaires* Saint-Luc (UCLouvain) and LabMCT at the Queen Astrid Military Hospital. Among them, two orthopedic bacterial strains (referred to as P2 and P3) were selected for their fitness to perform phage isolation protocols (no signs of spontaneous phage induction and reproducible growth in laboratory conditions) in addition to *P. aeruginosa* strains CN573 (initially developed at the Eliava Institute, Georgia, as a production host for *P. aeruginosa* phages) and *P. aeruginosa* PAO1 (reference strain used in biofilm models).

In the first sampling round, hospital sewages were chosen as the most optimal sampling sources to isolate orthopedically relevant phages. Sixteen sewage samples from eight different Belgian Hospital were used on top of water samples from six different Belgian and Dutch environmental sources (rivers, lakes, or ponds). Two samples collected at a biological water purification plant were also processed. Additional samples, such as nasal swabs, nasal washes, wound compresses, and gauzes from an orthopedic patient with a 2-year recurrent infection, were also included. A full list of the used samples is provided in Table S1.

All samples were first centrifuged at 6,000 × *g* for 10 min at 4°C and filtered through a 0.45 µm filter (Millex-HP, Merck Millipore Ltd) to remove residual bacteria and other waste products. Subsequently, 200 mL was subjected to an enrichment protocol (86). In short, samples were incubated overnight at 37°C with a 1 mL pre-culture mixture of *P. aeruginosa* strains PAO1, CN573, P2, and P3 in combination with 20 mL of both 10× concentrated Tryptic Soy Broth and LB (Lysogeny Broth) media to stimulate bacterial growth in the mixture (86). The day after, 10 mL chloroform was added to deteriorate bacterial cell walls, liberating phage particles still residing in the host bacterial cells. Samples were subsequently stored at 4°C for at least 1 h. For each sample, 50 mL was collected and centrifuged once again at 6,000 × *g*, for 10 min at 4°C. Then, the supernatant was filtered (0.45 µm) and stored at 4°C for at least 1 h before performing spot tests (87).

In short, for the spot tests, 100 µL of a 1/10 diluted overnight culture of the four bacterial strains used during the enrichment protocol (PAO1, CN573, P2, and P3) were separately added to 3 mL of 0.6% LB agar (LBA) and poured on Petri plates with filled in with 1.5% LBA. After drying of the plates, 10–20 µL drops of the processed samples were spotted on this bacterial culture and subsequently incubated overnight at 32°C. After incubation, lysis zones (as well as small parts of bacterial culture) were cut from these Petri plates and incubated in liquid LB media for 3 h at 37°C after which 100 µL chloroform was added before performing a double agar overlay method (88) to visualize individual plaques. Next, phages were plaque purified at least eight times (plaques were picked-up, re-cultured, and re-incubated with their respective host bacteria). Individual phage clones were then propagated to high titers using an adapted double agar overlay technique after first identifying the most optimal multiplicity of infection (identifying a web-like pattern). For this, semi-solid LBA was recuperated after phage-bacterial incubation at web-like conditions (time and temperature dependent), centrifuged at 6,000 × *g* at 4°C for 20 min followed by filtration through 0.45 and 0.22 µm filters. All phage clones were subsequently stored at 4°C.

Isolated, purified, and propagated phages were evaluated based on plaque morphology and host range by means of spot tests on 32 different *P. aeruginosa* strains (listed in Table S2 and S3).

### DNA extraction and genome sequencing

First, 1 mL of phage samples were incubated for 30 min at 37°C with 20 U DNAse and 50 mg RNAse protease free (Thermoscientific, Waltham, MA) to remove any residual bacterial nucleotide remnants. Then, 62.5 µL of Proteinase K (included in the kit) was added to destroy the capsid of phage particles. DNA extractions were performed by using the Purelink Viral RNA/DNA Mini kit, while Illumina sequencing was performed for the three phages selected based on our initial screening. Phage annotation was performed by using both PHANOTATE and BV-BRC (Viral Bioinformatics Resource Center). The absence of depolymerase genes was confirmed by evaluating the annotated genomes by using the online PhageDPO tool in parallel with DePP (89, 90). When one or both of these tools indicated a predictive score higher than 50%, individual protein sequences were searched for pectate lyase domains by using HHpred (MPI Bioinformatics Toolkit) with the Pfam-A_v35 structural/domain database (91). None of these searches yielded positive results.

### Formation of a mono-species biofilm including *P. aeruginosa* reference strain PAO1

Overnight culture of *P. aeruginosa* strain PAO1 on non-selective TSA media was suspended in phosphate-buffered saline (PBS). McFarland OD was adjusted to 1.6–1.8 (1.07–4.92 × 10^8^ CFU/mL) and diluted 1/40 in RPMI media supplemented with 1% glucose (RPMG) and 10% FBS (Fetal Bovine Serum) in 96-well plates [VWR Tissue Culture Plates 96-well, flat bottom, surface treated, sterile (Eur. Ref. 734–2327, VWR, Radnor, PA)], as previously described by Ruiz-Sorribas et al. 2022 (92). This medium was selected as it was shown to ensure reproducible biofilm growth and better mimics the environment of an infection site compared to conventional bacterial culture media. Plates were incubated for 24 h at 37°C. Additionally, PAO1 biofilms were grown on titanium alloy coupons (Ti-6Al-4V ELI, diameter 12.7 mm, thickness 3.8 mm; BioSurface Technologies, Bozeman, MT) following the same protocol and with equivalent conditions but adapted to a 2 mL volume in 24-well plates (VWR Tissue Culture 24-well, flat bottom, surface treated, sterile).

### Biofilm treatment

For biofilms grown in 96-well plates, the medium was removed after 24 h of culture, residual biofilms were washed with PBS, and different treatments (phage and/or antibiotic) were applied in the same medium and incubated during 24 h. Treatments consisted of phages at a concentration of 10^9^ PFU/mL or antibiotics, including ciprofloxacin [potency, 89% (Bayer, Lerverkusen, Germany)], ceftazidime [potency, 72% (Panpharma, Luitré-Dompierre, France)], and meropenem [potency, 92% (Fresenius Kabi, Schelle, Belgium)] at concentrations of 1 or 10× MIC, or a combination of phages and one of these antibiotics.

For biofilms grown on titanium coupons, only combined (24 and 48 h) but also sequential application of bacteriophage PSP30 (10^7^ PFU/mL) followed by ciprofloxacin (100× MIC) were evaluated (24 h of incubation with the phage followed by 24 h with the antibiotic and vice versa).

As a preliminary to these experiments, MIC values were determined for all three antibiotics on planktonic PAO1 cultures following the CLSI guidelines but adjusted to RPMG media supplemented with 10% FBS. Their values were 0.125 mg/L, 0.5 g/L, and 1 mg/L for ciprofloxacin, meropenem, and ceftazidime, respectively.

### PAS analysis

#### Biomass evaluation

At the end of the treatments, the medium was removed manually, and biofilms were washed again with PBS and dried overnight at 60°C. The next day, a 0.5% [(vol/vol), final concentration 115 mg/L] crystal violet staining (Sigma-Aldrich, Saint-Louis, MO) was performed for 10 min after which biofilms were washed again to remove the excess dye. Solubilization of the dye was done by using 66% acidic acid solution (Merck KGaA, Darmstadt, Germany), and optical density measurement with spectrophotometry (SpectraMax Gemini XS microplate spectrophotometer; Molecular Devices LLC, San José, CA) was performed at 570 nm.

#### Selective plating and CFU counting

Individual wells were washed with PBS after which the tip of an inoculation loop was used to mechanically detach biofilms from the bottom of the plates. This washing procedure was considered adequate to eliminate phages, as no reduction in CFU numbers was observed between control biofilms or biofilms exposed to phages (see results). Contents of the wells were then resuspended with 200 µL PBS, and sonication was performed directly in the individual wells (30 s at 60% amplitude; Q700; QSonica, Newton, CT) to disaggregate the biofilm. After sonication, a dilution series was made for each treatment protocol (in triplicate), and 50 µL of multiple dilutions were plated on Pseudomonas Isolation Agar (PIA, Millipore, Sigma Aldrich) and incubated for 24 h at 37°C. CFU counts were performed for multiple wells in each plate, and mean estimates of viable cell count were calculated.

#### Biolog OmniLog assays

Biofilms were grown in 96-well plates under exactly the same conditions as already described above. After 24 h, the medium was removed, and biofilms were washed. Different treatment modalities of phages and/or antibiotics were added in RPMG media supplemented with 10% FBS. Tetrazolium redox dye A (Biolog, Carlsbad, CA) was diluted 100-fold in the prepared media mixture according to the manufacturer’s instructions and subsequently applied to biofilms. Plates were immediately incubated for 48 h at 37°C in an OmniLog (Biolog) device. Results were analyzed using Omnilog Data Analysis Software 1.7.

#### Scanning electron microscopy

SEM was performed for biofilms grown for 24 h on titanium alloy (Ti-6Al-4V) coupons after which they were treated with either phage PSP30 (10^7^ PFU/mL), ciprofloxacin (100× MIC), or a combination of both following a previously described procedure (93). In brief, after incubation with phages and/or ciprofloxacin, washing steps, and overnight drying at 60°C, biofilms were fixed with 2.5% glutaraldehyde (Sigma) in a sodium cacodylate buffer 0.1 M at pH 7.4 (Sigma) for 30 min. After washing with PBS, biofilms were dehydrated by consecutive incubations (20 min) in solutions with ascending ethanol (Merck) concentrations (30, 50, 70, 90, and 100% ethanol, with the latter performed three times). After drying, these coupons were coated with platinum particles (Pt) by using a sputtering device (Quorum Q150T S, Quorum Technologies, Laughton, UK) and visualized using an FEI XL30-FEG SEM (Hillsboro, OR) at a high vacuum with a 10 keV voltage.

## Data Availability

Data from Fig. 1 to 3 are available in the supplemental material (Data Set S1). The phage genomes were submitted to NCBI GenBank and are available under accession numbers OR538761, OR538762, and OR538763 for phages PSP2, PSP3, and PSP30, respectively.

## SUPPLEMENTAL MATERIAL

Data Set S1 spectrum.03219-23-s0001.xlsx Raw data for Figures 1 to 3.

Supplemental material spectrum.03219-23-s0002.pdf Tables S1 to S6; Figures S1 to S3.

ASM does not own the copyrights to Supplemental Material that may be linked to, or accessed through, an article. The authors have granted ASM a non-exclusive, world-wide license to publish the Supplemental Material files. Please contact the corresponding author directly for reuse.
