# Supplementary material for: Exploiting phage-antibiotic synergies to disrupt Pseudomonas aeruginosa PAO1 biofilms in the context of orthopedic infections
Source: Microbiol Spectr. 2023 Dec 12;12(1):e03219-23. doi: 10.1128/spectrum.03219-23 (PMC10783084; doi:10.1128/spectrum.03219-23)

Exploiting Phage-Antibiotic Synergies to Disrupt *Pseudomonas aeruginosa* PAO1 Biofilms in the context of Orthopedic Infections

Steven De Soir, Hortence Parée, Nur Hidayatul Nazirah Kamarudin, Jeroen Wagemans, Rob Lavigne, Annabel Braem, Maya Merabishvili, Daniel De Vos, Jean-Paul Pirnay, Françoise Van Bambeke

Supplementary Materials

Table S1: List of bacteriophages de novo isolated from a wide variety of environmental sources (strain used for propagation is indicated between brackets; phages overlined in yellow are those used in this study)

UZ Leuven (Gasthuisberg)	***Cliniques Universitaires St.-Luc***	***Queen Astrid Military Hospital***	***Vilvoorde (Park/pond)***
GHB G1 (P3)	SLCNA (CN573)	MPCNHe (CN573)	TF1P2 (P2)
GHB G2 (P3)	SLCNB (CN573)	MP2A (P2)	TF1P4 (1) (on CN573)
GHB GI (CN573)	SLCNC (CN573)	MP2B (P2)	TF1P4 (2) (on CN573)
GHB kl (CN573)	SLP2B (P2)	MP3 (P3)	TF1P5 (on CN573)
GHB Gk (PAO1)	SLP2H (P2)	MP4 (on CN573)	TF2P2 (P2)
GHB GIT (PAO1)	SLP2I (P2)	MP5 (on CN573)	TF2P5 (on CN573)
**GHB k1 (PAO1) = PSP2**	SLPAO1J (PAO1)		
GHB I (P2)	***UZ Pellenberg Unfiltered***	***Biological Water Purification (Leuven)***	***Nijmegen (pond)***
GHB GT (P2)	UZPO GH1 (P3)	WZPK2 I (P2) (1/2 & 2/2)	NMPAO1 (PAO1)
GHB G (PAO1)	UZPO GH2 (P3)	WZPK2 IC (P3)	NMP2 (P2)
GHB GH (PAO1)	UZPO k (PAO1)	WZPK2 GIK3 (PAO1)	
GHB GHk (PAO1)	UZPO kl (P2)	WZPK M (PAO1)	
GHB k12 (P2)	***UZ Pellenberg Filtered***	***Sint-Augustinus Antwerpen***	***Klein-Willebroek (pond)***
GHB k2 (PAO1)	UZPF G (CN573)	SAU I (CN573)	KWCN (CN573)
GHB K2 & Kl (CN573)	UZPF dt (P3)	SAU k (CN573)	
GHB G (CN573)			
GHB2 HCT (CN573)	***UZ Gent***	***UZ Brussels (Jette)***	***Park Bruxelles (pond)***
GHB2 kH1 (P2)	**UZGP2Hc (P2)= PSP30**	UZB1 (P2)	UMA (CN573)
GHB2 kt1 (P2) (1/2 & 2/2)	UZGP2Hi (P2)	**UZB2 I (CN573) = PSP3**	
GHB2 GT2 (P2)	UZGP2T (P2)	***Patient compresses & wound gauze***	***Mastvest (pond Antwerp)***
GHB2 GH (P3)	UZGP3k (P3)	PC (PAO1)	Q (PAO1)
GHB2 IC (P3)	UZGCN (CN573)	PC (CN573)	

Table S2*: P.aeruginosa* strains from the LMG collection (https://bccm.belspo.be/about-us/bccm-lmg) at the LabMCT of the Queen Astrid Military Hospital used during host range evaluation:

LMG number	Strain	Location	Country	Year	Source	fAFLP	Serotype
25162	Jp1200	Pacific Ocean (S2, 0m)	Japan	2003	Sea water (open ocean)	44	11
25166	IDEXXCanine6	Unknown (IDEXX)	UK	2004	Dog	22	NT
25167	IDEXXCanine3	Unknown (IDEXX)	UK	2004	Dog	14	NT
25168	Be136	Beverwijk	The Netherlands	1996	Sputum		3
25170	Tu61	Mediterranean Sea	Tunisia	2000	Sea water (coastal)	3	6
25171	Jp222	Pacific Ocean (S2, 0m)	Japan	2004	Sea water (open ocean)	15	6
25172	Be133	Beverwijk	The Netherlands	1996	Burn		NT
25174	Br641	Brussels	Belgium	1998	Hospital environment	1	12
25177	Br642	Brussels	Belgium	1998	Hospital environment	4	1
25179	W15 Apr 4	Woluwe river	Belgium	2002	River water		1
25180	LiA7/2007	Lisbon	Portugal	2007	Dog eye	16	1
25183	LiA179/2006	Lisbon	Portugal	2006	Dog eye	16	1
25186	IDEXXCanine4	Unknown (IDEXX)	UK	2004	Dog	42	10
25190	J80UH1 OS1	Jekyll Island	USA	2005	Turtle egg (exterior)	18	3
25191	J66UH5 F7	Jekyll Island	USA	2005	Turtle egg (interior)	19	3
25194	A14	Paris	France	1882-1918	Wound	31	11
25196	CPHL 8505	Unknown (NIMR)	UK	1950	Unknown		3
25198	DVL1758	Ghent	Belgium	2003	Shallow pond water		NT
25199	CPHL 11451	Kentucky	USA	1982	Unknown		12

Table S3: host Range evaluation of phage PSP2, PSP3 and PSP30 on orthopedic clinical isolates (left) and LMG collection *P.aeruginosa* strains (right)

Strain	**PSP2**	**PSP3**	**PSP30**
P2	√	√	LFW
P3	√	√	√
CN573	√	√	√
PAO1	√	√	√
Clin.1	LFW	√	√
Clin. 2	√	√	√
Clin. 3	√	-	√
Clin. 4	√	-	√
Clin. 5	√	√	√
Clin. 6	√	√	√
Clin. 7	√	√	√
Clin. 8	√	√	√
Clin. 9	√	√	√

Strain	**PSP2**	**PSP3**	**PSP30**
LMG 25162	√	LFW	√
LMG 25166	√	√	√
LMG 25167	√	-	-
LMG 25168	√	LFW	√
LMG 25170	√	√	√
LMG 25171	√	LFW	√
LMG 25172	√	√	√
LMG 25174	√	√	√
LMG 25177	√	LFW	√
LMG 25179	√	√	√
LMG 25180	√	√	√
LMG 25183	√	√	√
LMG 25186	√	√	√
LMG 25190	√	√	-
LMG 25191	√	LFW	√
LMG 25194	√	LFW	√
LMG 25196	√	√	√
LMG 25198	√	LFW	√
LMG 25199	√	LFW	√

Legend:

√ = Phage propagation observed

LFW = Lysis from without

- = No phage activity observed on the tested strain

Figure S1: Easyfig alignments of phages PSP2/PSP3/PSP30

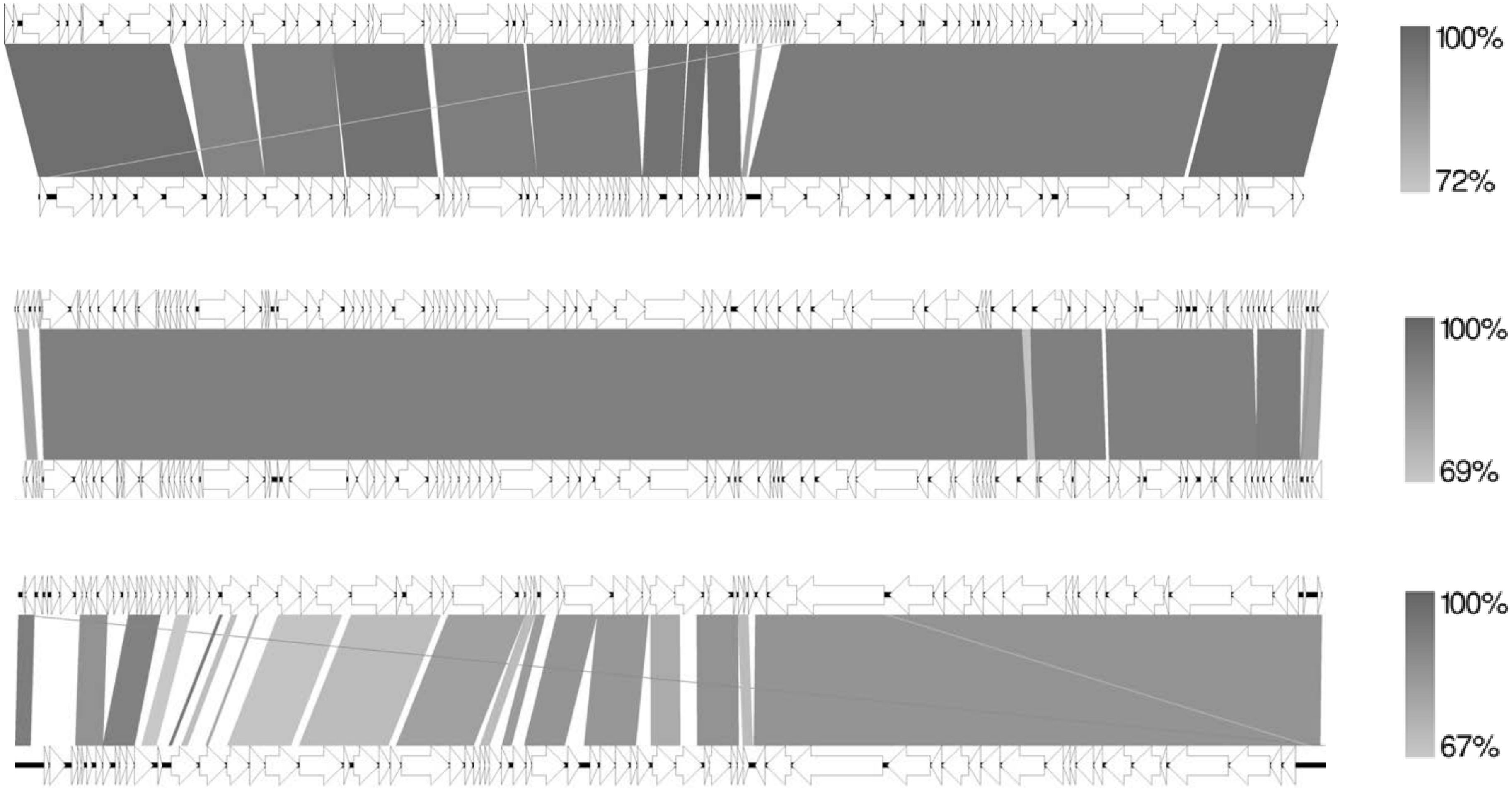

Alignments of de novo-isolated phages PSP2, PSP3, and PSP30 with their identified **homologous** viral counterparts (*Yuavirus, Pbunavirus* and *Bruynoghevirus* respectively from top to bottom).

Table S4: Synergetic profiles of the combined treatment of ciprofloxacin with phage PSP2, PSP3, PSP30 or the phage cocktail. Color codes indicate the level of interaction. **Green = synergy** (observed effect greater than the sum of the effect of both individual agents), **yellow = additive effect** (higher than the effect of any type of agent alone), **red = no effect** (not higher than the effect observed for any individual agent).

**1MIC**		
	**Biomass (% reduction)**	**CFUs (log reduction)**
Cipro	0.094	1.71
Phage 2	0.082	0.60
Combined	***0.175***	***2.31***
Observed effect	**0.153**	**2.48**
Cipro	0.094	1.71
Phage 3	0.073	0.48
Combined	***0.166***	***2.18***
Observed effect	**0.247**	**2.69**
Cipro	0.094	1.71
Phage 30	0.068	-0.16
Combined	***0.161***	1.54
Observed effect	**0.238**	**2.97**
Cipro	0.094	1.71
Phage Cocktail	0.054	1.23
Combined	***0.148***	2.93
Observed effect	**0.239**	***3.64***

**10MIC**		
	**Biomass (% reduction)**	**CFUs (log reduction)**
Cipro	0.229	2.30
Phage 2	0.124	1.11
Combined	***0.354***	***3.41***
Observed effect	**0.230**	**3.56**
Cipro	0.229	2.30
Phage 3	0.086	0.63
Combined	***0.316***	***2.93***
Observed effect	**0.297**	**2.91**
Cipro	0.229	2.30
Phage 30	0.067	0.18
Combined	***0.297***	***2.48***
Observed effect	**0.255**	**3.36**
Cipro	0.229	2.30
Phage Cocktail	0.085	1.66
Combined	***0.315***	3.95
Observed effect	**0.285**	**3.08**

Table S5: Synergetic profiles of the combined treatment of meropenem with phage PSP2, PSP3, PSP30 or the phage cocktail. Color codes indicate the level of interaction. **Green = synergy** (observed effect greater than the sum of the effect of both individual agents), **yellow = additive effect** (higher than the effect of any type of agent alone), **red = no effect** (not higher than the effect observed for any individual agent).

**1MIC**		
	**Biomass (% reduction)**	**CFUs (log reduction)**
Mero	0.024	-0.61
Phage 2	0.059	1.03
Combined	***0.083***	***0.42***
Observed effect	**0.132**	**1.85**
Mero	0.024	-0.61
Phage 3	0.088	0.16
Combined	***0.113***	***-0.45***
Observed effect	**0.113**	**1.33**
Mero	0.024	-0.61
Phage 30	0.034	-0.23
Combined	***0.058***	-0.84
Observed effect	**0.101**	**0.52**
Mero	0.024	-0.61
Phage Cocktail	0.085	1.78
Combined	0.110	1.17
Observed effect	***0.209***	***1.88***

**10MIC**		
	**Biomass (% reduction)**	**CFUs (log reduction)**
Mero	0.057	0.73
Phage 2	0.071	**1.37**
Combined	***0.128***	***2.10***
Observed effect	**0.262**	**2.64**
Mero	0.057	0.73
Phage 3	0.108	0.35
Combined	***0.165***	***1.08***
Observed effect	**0.299**	**1.53**
Mero	0.057	0.73
Phage 30	0.076	-0.22
Combined	***0.133***	***0.51***
Observed effect	**0.221**	**1.78**
Mero	0.057	0.73
Phage Cocktail	0.106	1.93
Combined	***0.163***	2.66
Observed effect	**0.284**	**2.89**

Table S6: Synergetic profiles of the combined treatment of ceftazidime with phage PSP2, PSP3, PSP30 or the phage cocktail. Color codes indicate the level of interaction. **Green = synergy** (observed effect greater than the sum of the effect of both individual agents), **yellow = additive effect** (higher than the effect of any type of agent alone), **red = no effect** (not higher than the effect observed for any individual agent).

	**1MIC**		**10MIC**	
	**Biomass (% reduction)**	**CFUs (log reduction)**	**Biomass (% reduction)**	**CFUs (log reduction)**
Cefta	-0.041	1.36	-0.020	1.28
Phage 2	-0.022	0.82	0.110	**0.36**
Combined	***-0.063***	***2.18***	***0.090***	***1.64***
Observed effect	**0.041** (green)	**0.83** (red)	**0.112** (green)	**4.30** (green)
Cefta	-0.041	1.36	-0.020	1.28
Phage 3	-0.018	1.46	0.070	1.26
Combined	***-0.059***	***2.82***	***0.050***	***2.54***
Observed effect	**0.077** (green)	**2.32** (yellow)	**0.166** (green)	**4.59** (green)
Cefta	-0.041	1.36	-0.020	1.28
Phage 30	0.067	0.34	0.058	0.40
Combined	***0.026***	1.70	***0.038***	***1.68***
Observed effect	**0.061** (green)	**2.25** (green)	**0.106** (green)	**2.57** (green)
Cefta	-0.041	1.36	-0.020	1.28
Phage Cocktail	0.029	1.19	0.010	0.66
Combined	-0.012	2.55	***-0.010***	1.94
Observed effect	***0.180*** (green)	***1.80*** (green)	**0.125** (green)	**1.92** (yellow)

Figure S2: SEM images of PAO1 biofilms grown on titanium coupons subjected to 48h treatment protocols. Forty-eight-hour continuous treatment of PAO1 biofilms grown on titanium coupons including a positive control or untreated biofilm **(panel A)** compared with treatment modalities consisting of either phage PSP30 **(panel B)**, ciprofloxacin **(panel C)** or a combined phage-antibiotic treatment **(panel D)**.

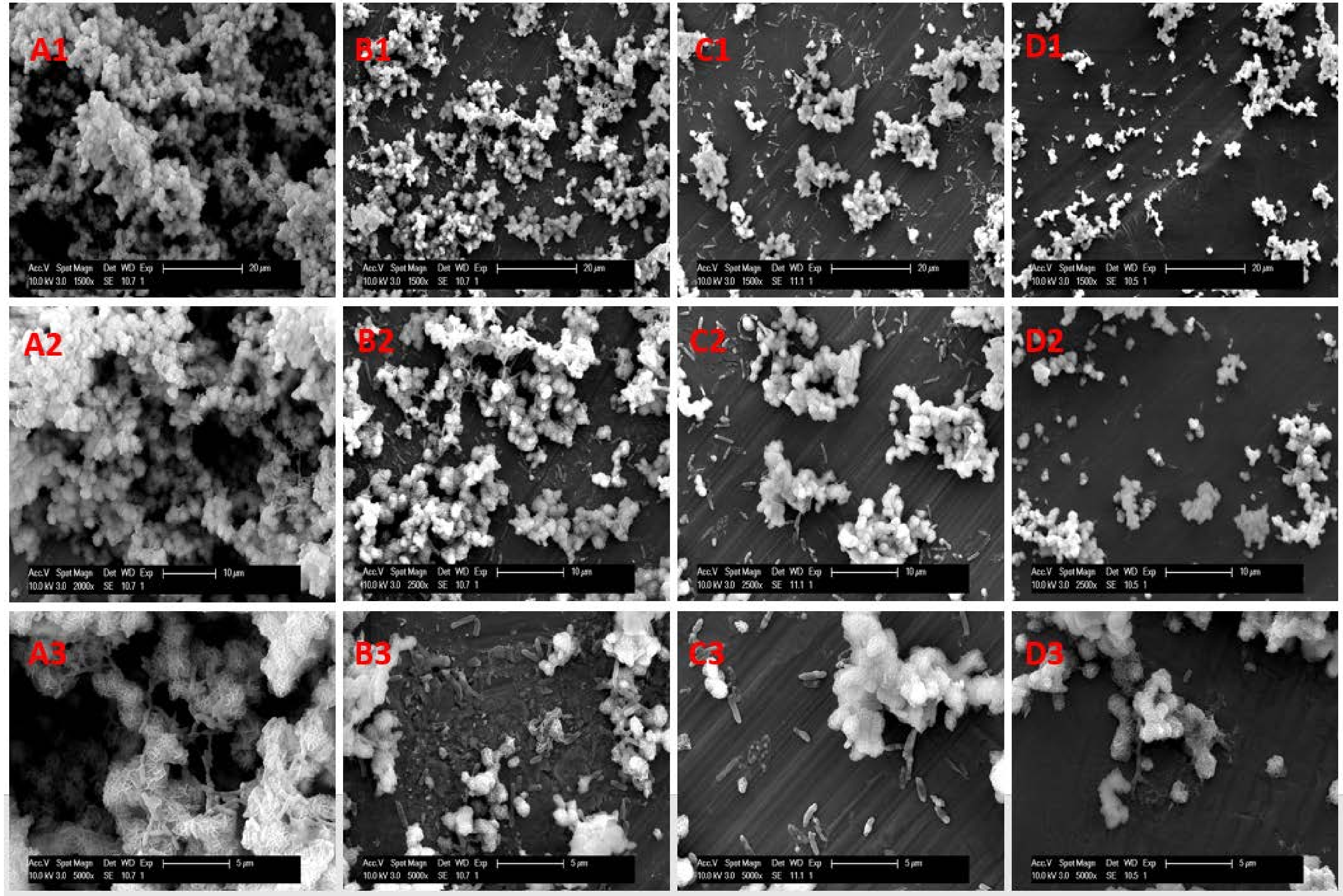

Figure S3: SEM images of PAO1 biofilms grown on titanium coupons & subjected to continuous or sequential phage-antibiotic therapy. Sequential treatment **(A= phage → AB, & B = AB→ phage) versus Combined treatment (C) of phage PSP30 and ciprofloxacin** respectively on PAO1 biofilms formed on titanium coupons. **48h Combined treatment (C)** clearly showing **limited to no viable PAO1 cells residing on the titanium coupons** in between biofilm matrix residues when compared to any type of sequential therapy **(A & B).**

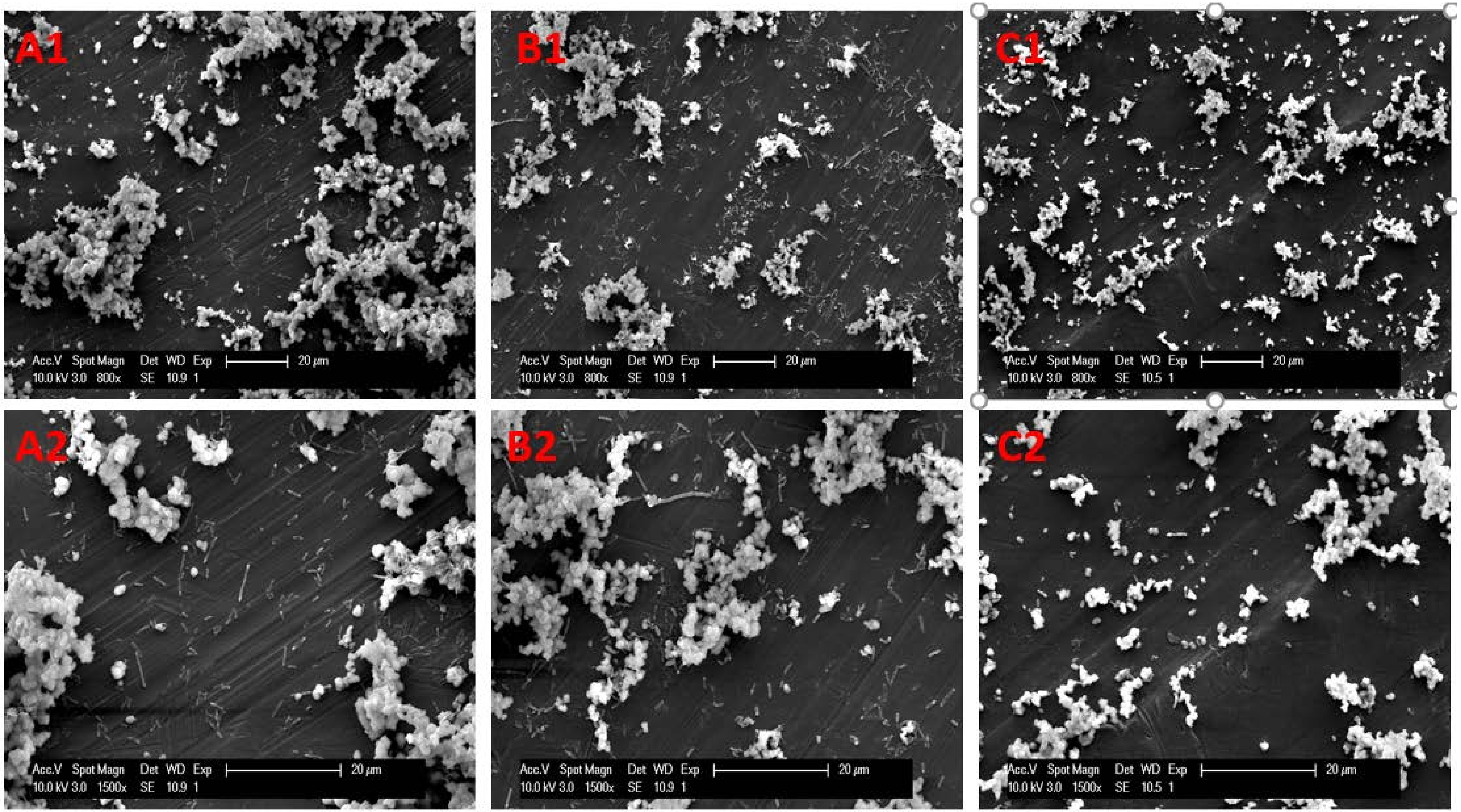

Supplement: Supplemental material — Tables S1 to S6; Figures S1 to S3. [file spectrum.03219-23-s0002.pdf]
